# Molecular determination of antimicrobial resistance in *Escherichia coli* isolated from raw meat in Addis Ababa and Bishoftu, Ethiopia

**DOI:** 10.1186/s12941-017-0233-x

**Published:** 2017-08-15

**Authors:** Yohannes Equar Messele, Reta Duguma Abdi, Shimels Tikuye Yalew, Desiye Tesfaye Tegegne, Bezina Arega Emeru, Gebremeskel Mamu Werid

**Affiliations:** 1 Ethiopian Institute of Agricultural Research, National Agricultural Biotechnology Research Center, P.O.Box 31, Holeta, Ethiopia; 2 College of Veterinary Medicine and Agriculture, Addis Ababa University, P.O.Box 34, Bishoftu, Ethiopia; 3 University of Tennessee Institute of Agriculture Department of Animal Science, Rivers Drive, Knoxville, TN USA

**Keywords:** Antibiotic resistance, *Escherichia coli*, Meat, Ethiopia

## Abstract

**Background:**

Consumption of meat contaminated by *E. coli* causes a serious illness and even death to affected individuals. Recently the emerging of antibiotic resistant foodborne *E. coli* poses serious public health risks worldwide. However, little is known about the antibiotic resistance profile of *E. coli* in Ethiopia. This study aimed to determine the prevalence and Antimicrobial resistance (AMR) status of *E. coli* isolated from different type of meat.

**Methods:**

Overall 292 samples were collected from December 2015 to April 2016 from slaughterhouses to determine the prevalence and AMR of *E. coli* isolated from raw beef, mutton, chevon and chicken meat from Addis Ababa and Bishoftu, Ethiopia. The isolates were screened for AMR against commonly used antibiotics circulating in the Ethiopian market. Both phenotypic and genotypic approach were employed for AMR detection using disc diffusion test and PCR respectively.

**Results:**

The prevalence of *E.* *coli* was 63 (21.6%), indicating one sample in every five samples harbors *E. coli*. Among these, the highest *E. coli* isolates was observed in chicken meat samples (37.0%; 27), followed by mutton (23.3%; 17), chevon (20.6%; 15) and beef (5.5%; 4). Results of disk diffusion test on the 63 isolates showed that only 4.8% of them were not resistance to all antimicrobials tested. Multiple drug resistance (resistance to ≥3 drugs) was 46.0%. Significantly high resistance to ampicillin (71.4%) and tetracycline (47.6%) was observed. Identification of genes associated with AMR was also done using PCR. The prevalence of *E.* *coli* isolates harboring resistance gene responsible for tetracycline *(tet*(*A*)), beta lactams *(blaCMY)* and sulphanamide (*sulI)* antibiotics were found 65.1, 65.1 and 54.0%, respectively. Twenty-five out of the 63 (39.7% %) *E.* *coli* isolates have got antimicrobial resistance gene to three or more classes of drugs. The associations of antimicrobial resistance phenotypes and resistance genes was also determined. The detection of resistance trait against tetracycline, sulphametazole and chloramphenicol measured either phenotypically or genotypically were high.

**Conclusions:**

The rising levels of resistance *E. coli* to multiple antimicrobial dictate the urgent need to regulate and monitor antimicrobial use in both animals and humans.

## Background

Meat and meat products serve as important source of proteins for humans. However, recently the emerging antibiotic resistant foodborne pathogens combined with the injudicious use of antibiotics in animals bears considerate public health threats worldwide. Usually, meat and meat products gets contaminated by pathogens during animal slaughter and food processing. *Escherichia coli (E. coli)* is the frequently isolated foodborne pathogens from meat and meat products. Meat can be contaminated by *E. coli* during animal slaughter due to unhygienic slaughter practices, through airborne, rodents, insects, and other animals [1]. Consumption of meat contaminated by *E. coli* causes a serious illness and even death to affected individuals [2].

Recently published reports indicated that the *E. coli* strains isolated from contaminated meat and meat products have become resistant to commonly used antibiotics. This is mainly due to injudicious usage of antibiotics in both humans and animals [3]. The wide spread and imprudent use of antibiotics in food animals is thought to be accountable for the emergence and wider spreading of antimicrobial resistant (AMR) bacteria in humans [3, 4]. In humans, positive selection for drug resistant bacteria have also been reported in the normal microflora of exposed individuals or populations. This indicates that antibiotic resistance can be developed in both commensal and pathogenic bacterial strains and can even be transferred to other bacterial strains, including other pathogenic and environmental bacteria [5].

Consumption of contaminated and/or uncooked meat poses the risks of acquiring foodborne *E. coli* strains [6] causing a serious public health concern. These bacterial strains easily harbors antibiotic resistant genes from one another. This is because genes encoding AMR determinants that are carried on mobile genetic elements such as plasmids and transposons of some bacterial strains could be transferred to other bacteria strains during contact [7], causing a threat to cure acute infections in man and animals. In Ethiopia, a review done by Alemu et al. [8] showed that several pathogen have established resistance against oxytetracycline drugs. Similarly, oxytetracycline and penstrep are the main prescribed antibiotics in Ethiopia [8, 9]. So far, no study has been carried out to identify the antibiotic resistance genes of *Escherichia coli* in Ethiopia.

In this paper, we explain the phenotypic and genotypic sources of MDR in *Escherichia coli* isolates recovered from different type of meat samples taken from Addis Ababa city abattoir and Alema farm poultry slaughter slab, Ethiopia. This enquiry pursues to deliver useful evidence on the prevalence and AMR profile of *E. coli* isolated from beef, mutton, chevon and chicken meat.

## Methods

### Sample collection and isolation of *E. coli*

The study was carried out from December 2015 to April 2016 to collect meat sample from Addis Ababa city abattoir enterprise and Alema farm slaughter slab found in Bishoftu town, Ethiopia. A total of 292 meat sample including beef meat (n = 73), sheep meat (n = 73), goat meat (n = 73) were collected from Addis Ababa municipal abattoir and chicken meat (n = 73) from Alema farm slaughter slab located in Bishoftu city. Sample were collected by swabbing from carcass gluteal muscle and inserted into 10 ml peptone water containing test tube. The samples were taken following the guidelines of the International Organization for Standardization [10]. The sample were transported to National Agricultural Biotechnology Research Center in an ice box for further processing. The *E. coli* isolates were identified by using standard bacteriological methods, comprising of colony structure determination, using different culture media and biochemical tests as previously indicated [11]. Isolates were preserved at −80 °C in a trypticase soy broth with 10% glycerol for further analysis.

### Antimicrobial susceptibility testing

Antibiotic sensitivity was determined by disc diffusion method according to the guidelines of the Clinical and Laboratory Standards Institute [12]. Bacterial suspension was prepared by adding 2–4 colonies to a 5 ml tube containing 0.9% normal saline (NaCl), to achieve absorbance of 0.17–0.18 at wavelength of 600 nm (equivalent to 0.5 McFarland standards) [13]. The suspension was spread onto Mueller–Hinton agar media (HIMEDIA, India) using a sterile cotton swab, and the antibiotic disc (Oxoid, UK) were placed on the top of the agar plate. The inoculated plates were incubated aerobically at 37 °C for 18–24 h, after which all *E. coli* isolates were tested for susceptibility using the following antimicrobial discs and their corresponding concentrations: tetracycline (30 μg/disk); streptomycin (10 μg/disk); chloramphenicol (30 μg/disk), sulfamethoxazole–trimethoprim (25 μg/disk), gentamycin (10 μg/disk); ampicillin, (10 μg/disk); erythromycin (15 μg/disk); by the Kirby-Bauer disk diffusion method [14].

### DNA extraction and PCR amplification of resistance genes

Bacterial strains were grown overnight in nutrient agar (HIMEDIA, India) at 37 °C. A loop full of the colonies was added to 100 μl of sterile water. Bacterial DNA was extracted by boiling a bacterial suspension in water. After boiling the suspension for 13 min, the suspension were frozen for 5 min in ice and centrifuged at 14,000 rpm for 15 min to pellet the cell debris [15]. The supernatant from the centrifuged tubes was transferred to new 1.5 ml clean plastic tube and used as a template for PCR amplification. The purified DNA were detected by electrophoresis in 1.5% agarose gel and then kept at −20 °C for further use. Primers for AMR genes such as streptomycin (*aadA1*), tetracycline [*tet*(A)], gentamicin [*aac*(*3*)-*IV*], sulfonamides (*sul1*), beta-lactams (*bla*SHV, *bla*CMY), erythromycin [*ere*(A)] and chloramphenicol (*catA1, cmlA*) were used from published article. The specific primer sequences (Bioneer, South Korea) and the estimated size of the amplified products for different resistance gene coding regions are found in Table 1. Amplification of antimicrobial resistance gene from *E.* *coli* isolates were performed as described by Fode et al. [16]. The amplification products were then separated by electrophoresis on a 1.5% agarose gel stained with gel red (Biotium Inc, USA) as described by Huang et al. [17] and visualized using UV illumination. A 100 bp DNA molecular marker (Bioneer, South Korea) were used to determine the size of the PCR product.

**Table 1:** Primers used for detection of antimicrobial resistant genes in *Escherichia coli* isolates

Drug type	Antimicrobial resistance genes	Primers	Sequence 5′–3′	Amplicon size (Bp)	References
Streptomycin	Adenylyl transferases (*aadA1*)	*aadA1*F	TATCCAGCTAAGCGCGAACT	447	[18]
		*aadA1*R	ATTTGCCGACTACCTTGGTC		
Gentamicin	Aminoglycoside acetyltransferases (*aac*(*3*)-*IV*)	*aac*(*3*)-*IV*F	CTTCAGGATGGCAAGTTGGT	286	
		*aac*(*3*)-*IV*R	TCATCTCGTTCTCCGCTCAT		
Sulfonamide	Dihydropteroate synthase (*sul1*)	*sul1*F	TTCGGCATTCTGAATCTCAC	822	
		*sul1R*	ATGATCTAACCCTCGGTCTC		
Beta-lactams	*β*-lactamase encoding penicillin resistance *(bla*_SHV)_	*bla*_SHV_F	TCGCCTGTGTATTATCTCCC	768	
		*bla*_SHVR_	CGCAGATAAATCACCACAATG		
	*β*-lactamase encoding cephalosporin resistance (*bla*_CMY_)	*bla*_CMY_F	TGGCCAGAACTGACAGGCAAA	462	
		*bla*_CMY_R	TTTCTCCTGAACGTGGCTGGC		
Erythromycin	Erythromycin esterase (*ere*(A))	*ere*(A)F	GCCGGTGCTCATGAACTTGAG	419	
		*ere*(A)R	CGACTCTATTCGATCAGAGGC		
Chloramphenicol	Acetyltransferases (*catA1)*	*catA1*F	AGTTGCTCAATGTACCTATAACC	547	
		*catA1*R	TTGTAATTCATTAAGCATTCTGCC		
	Transporter resistance (*cmlA)*	*cmlA*F	CCGCCACGGTGTTGTTGTTATC	698	
		*cmlA*R	CACCTTGCCTGCCCATCATTAG		
Tetracycline	Efflux pump resistance (*tet*(A))	*tet*(A)F	GGTTCACTCGAACGACGTCA	577	[19]
		*tet*(A)R	CTGTCCGACAAGTTGCATGA		

### Statistical analysis

The prevalence of *E. coli* infection was quantified and compared among meat samples of different livestock species. Prevalence of AMR was quantified along with resistance patterns. The data were analyzed by using SPSS software version 20 and P value was calculated using Chi square and Fisher’s exact tests to determine any significant correlation. P value less than 0.05 was considered statistically significant.

## Results

### Prevalence of *E. coli*

In this study, a total of 63 (21.6%) *E. coli* isolates were identified from the 292 raw meat samples examined (Table 2). Of these positive cases, chicken meat had the highest (37.0%) whilst beef meat had the lowest prevalence (5.5%) of *E. coli*. A significance difference in *E. coli* prevalence (P < 0.05) was observed among meat samples of different livestock species. In this regard, meat of chevon 2.8 times, mutton 3.2 times and chicken meat 5.7 times were more infected than beef meat by *E. coli* indicating a difference in *E. coli* infection risk to humans based on meat type.

**Table 2:** Prevalence of *E. coli* in meat samples of different livestock species

Meat sample	Total sample tested	No. of positives	Prevalence (%)	OR	P value
Chicken	73	27	37.0	Reference	<0.001
Mutton	73	17	23.3	0.51	
Chevon	73	15	20.6	0.44	
Beef	73	4	5.5	0.10	
Total	292	63	21.6		

### Phenotypic antibiotic susceptibility test

The distribution of Antimicrobial resistance (AMR) among the *E.* *coli* isolates from meat samples of four livestock species was shown in Table 3. The lists of AMR was Ampicillin (71.4%), tetracycline (47.6%), erythromycin (42.8%), streptomycin (36.5%), sulfamethoxazole–trimethoprim (34.9%), chloramphenicol (23.8%) and gentamycin (4.8%). Among the *E.* *coli* isolates identified from meat of different food animals, resistance to ampicillin was highest in chevon (86.7%), followed by chicken (70.4%), mutton (64.7%) and beef meat (50%) as presented in Table 3.

**Table 3:** Antimicrobial sensitivity test of *E. coli* isolates (n = 63) sampled from meat of different livestock species

	E	AMP	GN	S	TE	Sxt	C
Beef (n = 4)							
R (%)	0	2 (50.0)	0	2 (50.0)	2 (50.0)	1 (25.0)	1 (25.0)
I (%)	2 (50.0)	1 (25.0)	0	1 (25.0)	0	0	1 (25.0)
S (%)	2 (50.0)	1 (25.0)	4 (100)	1 (25.0)	2 (50.0)	3 (75)	2 (50.0)
Mutton (n = 17)							
R (%)	9 (53.0)	11 (64.7)	2 (11.8)	7 (41.2)	4 (23.5)	3 (17.6)	2 (11.8)
I (%)	7 (41.2)	3 (17.6)	1 (5.9)	3 (17.6)	2 (11.8)	3 (17.6)	3 (17.6)
S (%)	1 (5.9)	3 (17.6)	14 (82.3)	7 (41.2)	11 (64.7)	11 (64.7)	12 (70.6)
Chevon (n = 15)							
R (%)	7 (46.7)	13 (86.7)	1 (6.7)	1 (6.7)	3 (20)	1 (6.7)	1 (6.7)
I (%)	8 (53.3)	0	1 (6.7)	5 (33.3)	2 (13.3)	2 (13.3)	0
S (%)	0	2 (13.3)	13 (86.7)	9 (60)	10 (66.7)	12 (80.0)	14 (93.3)
Chicken (n = 27)							
R (%)	11 (40.7)	19 (70.4)	0	13 (48.1)	21 (77.8)	17 (63.0)	11 (40.7)
I (%)	10 (37.0)	3 (11.1)	7 (26.0)	6 (22.2)	4 (14.8)	3 (11.0)	5 (18.5)
S (%)	6 (22.2)	5 (18.5)	20 (74.0)	8 (29.6)	2 (7.4)	7 (26.0)	11(40.7)
Overall							
R (%)	27 (42.9)	45 (71.4)	3 (4.8)	23 (36.5)	30 (47.6)	22 (34.9)	15 (23.8)
I (%)	27 (42.9)	7 (11.1)	9 (14.3)	15 (23.8)	8 (12.7)	8 (12.7)	9 (14.3)
S (%)	9 (14.3)	11 (17.5)	51 (81.0)	25 (39.7)	25 (39.7)	33 (52.4)	39 (61.9)

*S* sensitive, *I* intermediate, *R* resistant, *E* erythromycin, *AMP* ampicillin, *GN* gentamycin, *S* streptomycin, *TE* tetracycline, *Sxt* sulfamethoxazole–trimethoprim, *C* chloramphenicol

Of 63 isolates, only 3 isolates (4.8%) were pan-sensitive and the rest were single drug resistance (12 isolates; 20.4%), double drug resistance (19; 30.2%) and multidrug (≥3 or more drugs) resistance (29; 46.0%). Two isolates demonstrated resistance to six different drugs. The AMR patterns and AMR frequencies were shown in Table 4. The most common AMR pattern found in the multiresistant isolates was ampicillin, tetracycline and erythromycin which jeopardizes the use of these drugs in the study area.

**Table 4:** Antimicrobial resistance pattern of *E. coli* isolates from meat samples of different livestock species

Number of antibiotic classes (number of isolates; %)	AMR pattern	Number of isolates (%)
No resistance		3 (4.7)
One (n = 13; 20.6%)	AMP	10 (15.9)
	E	1 (1.6)
	TE	1 (1.6)
	C	1 (1.6)
Two (n = 19; 30.2%)	AMP*C	1 (1.6)
	AMP*S	1 (1.6)
	AMP*Sxt	1 (1.6)
	AMP*TE	4 (6.3)
	E*AMP	6 (9.5)
	E*S	1 (1.6)
	E*TE	3 (4.8)
	TE*Sxt	1 (1.6)
Three (n = 11; 17.5%)	AMP*Sxt*C	1 (1.6)
	AMP*TE*C	1 (1.6)
	AMP*TE*Sxt	3 (4.8)
	E*AMP*S	1 (1.6)
	E*AMP*Sxt	1 (1.6)
	S*Sxt*C	1 (1.6)
	S*TE*Sxt	3 (4.8)
Four (n = 9; 14.3%)	AMP*S*TE*C	1 (1.6)
	AMP*S*TE*Sxt	1 (1.6)
	E*AMP*GN*S	3 (4.8)
	E*AMP*S*TE	2 (3.4)
	E*AMP*Sxt*C	1 (1.6)
	E*AMP*TE*C	1 (1.6)
Five (n = 7; 11.1%)	AMP*S*TE*Sxt*C	2 (3.4)
	E*AMP*S*TE*Sxt	2 (3.4)
	E*S*TE*Sxt*C	3 (4.8)
Six (n = 2; 3.2%)	E*AMP*S*TE*Sxt*C	2 (3.4)

*E* erythromycin, *AMP* ampicillin, *GN* gentamicin, *S* streptomycin, *TE* tetracycline, *Sxt* sulfamethoxazole–trimethoprim, *C* chloramphenicol

### Molecular detection of antimicrobial resistance

**Fig. 1:**
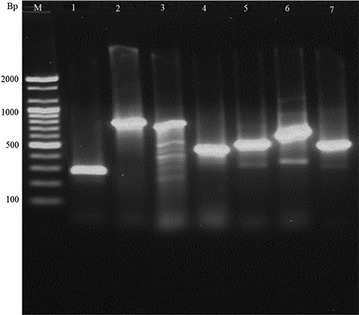
PCR detection of *E. coli* AMR gene as visualized using agarose gel electrophoresis. *Bp* base pair, *M* molecular weight standard, *lane 1 aac(3)*-*IV* (286 Bp), *lane 2 sul1* (822 Bp), *lane 3 bla*_*SHV*_ (768 Bp), *lane 4 bla*_*CMY*_ (462 Bp), *lane 5 catA1* (547 Bp), *lane 6 cmlA* (698 Bp), *lane 7 tet(A)* (577 Bp)

All of the 63 *E.* *coli* isolates were confirmed by PCR to determine the existence of any AMR gene. The erythromycin esterase gene *ere*(*A*) and the aminoglycoside adenylyl transferases genes *aadA1* were not identified in any of the 63 *E. coli* isolates (Fig. 1). The *ere*(A) gene confers resistance to erythromycin whilst aadA1 gene confer resistance to streptomycin. The most commonly detected AMR genes were *blaCMY* (65.1%), *tetA* (65.1%) and *sul1* (54.0%). The distribution of resistance genes among *E.* *coli* isolates are summarized in Table 5. The *bla*_*SHV*_, β-lactamase gene and *bla*_*CMY*_ genes were identified in 4.8 and 65.1% of the isolates, respectively. The *aac*(*3*)-*IV* gene which codes resistance against gentamycin was found also in 14.3% of isolates.

**Table 5:** Distribution of AMR genes in *E.* *coli* isolates (n = 63) using PCR test

Meat type	Number of isolates with AMR gene (%)								
	*aadA1*	*aac(3)*-*IV*	*sul1*	*bla*_SHV_	*bla*_CMY_	*ere*(A)	*catA1*	*cmlA*	*tet*(A)
Beef (n = 4)	0	0	1 (25.0)	0	0	0	1 (25.0)	0	0
Mutton (n = 17)	0	0	5 (29.4)	0	11 (64.7)	0	1 (5.9)	0	11 (64.7)
Chevon (n = 15)	0	1 (6.7)	9 (60.0)	0	14 (93.3)	0	0	0	5 (33.3)
Chicken (n = 27)	0	8 (29.6)	19 (70.4)	3 (11.1)	16 (59.2)	0	3 (11.1)	7 (26.0)	25 (92.6)
Total	0	9 (14.3)	34 (54.0)	3 (4.8)	41 (65.1)	0	5 (8.0)	7 (11.1)	41 (65.1)

*aadA1* streptomycin, *aac(3)*-*IV* gentamicin, *sul1* sulfonamide, *bla*_*SHV*_ and *bla*_*CMY*_ beta lactams, *ere(A)* erythromycin, *catA1* and *cmlA* chloramphenicol, *tet(A)* tetracycline

Overall, 39.7% (25/63) of isolates harbored resistance gene responsible to three or more drugs (Table 6). The most commonly detected genes were *tetA,* sul1, and *bla*_*CMY*_. The most common pattern detected was *sulI* and *tet*(*A*) together (n = 23; 36.5%) followed by *bla*_*CMY*_ and *tetA* (n = 22; 35.0%) and bla_CMY_ and *sulI* (n = 20; 31.7%).

**Table 6:** Multiple antimicrobial resistance gene patterns of *E. coli* isolates

Number of AMR gene (%)	Resistance gene patterns	Number of isolates (%)
No resistance		7 (11.1)
One (n = 11; 17.5%)	*bla*_CMY_	6 (9.5)
	*tet*(A)	3 (4.7)
	*sul1*	1 (1.6)
	*catA1*	1 (1.6)
Two (n = 20; 31.8%)	*aac(3)*-*IV* * *bla*_CMY_	1 (1.6)
	*aac(3)*-*IV* * *tet*(A)	1 (1.6)
	*cmlA* * *tet*(A)	1 (1.6)
	*bla*_CMY_ * *tet*(A)	7 (11.1)
	sul1* *bla*_CMY_	5 (7.9)
	sul1* *catA1*	1 (1.6)
	sul1* *tet*(A)	4 (6.3)
Three (n = 15; 23.8%)	sul1* *bla*_CMY_ * *tet*(A)	10 (15.9)
	sul1* *cmlA* * *tet*(A)	2 (3.2)
	*aac(3)*-*IV* * sul1* *tet*(A)	1 (1.6)
	*bla*_CMY_ * *cmlA* * *tet*(A)	1 (1.6)
	*bla*_CMY_ * catA1* *tet*(A)	1 (1.6)
Four (n = 6; 9.5%)	sul1* *bla*_CMY_ * catA1* *tet*(A)	2 (3.2)
	*aac(3)*-*IV* * sul1* *bla*_CMY_ * *tet*(A)	2 (3.2)
	sul1* *bla*_CMY_ * *cmlA* * *tet*(A)	1 (1.6)
	*sul1* * *bla*_SHV_ * *bla*_CMY_ * *tet*(A)	1 (1.6)
Five (n = 4; 6.3%)	*aac(3)*-*IV* * sul1* *bla*_CMY_ * *cmlA* * *tet*(A)	2 (3.2)
	*aac(3)*-*IV* * sul1* *bla*_SHV_ *bla_CMY_* *tet*(A)	2 (3.2)

*aadA1* streptomycin, *aac(3)*-*IV* gentamicin, *sul1* sulfonamide, *bla*_*SHV*_ and *bla*_*CMY*_ beta lactams, *ere(A)* erythromycin, *catA1* and *cmlA* chloramphenicol, *tet(A)* tetracycline

### Relationship between drug resistance by disc diffusion and PCR methods

The most commonly drugs to which isolates demonstrated resistance by disk diffusion method were consistent with the most common resistance genes detected (Table 7). Tetracycline, sulfamethoxazole and chloramphenicol were top three drugs recognized as being the most common for resistance measured either disk diffusion or molecular method. However, the phenotypic and genotypic resistance characterization of the isolates to erythromycin, streptomycin and gentamycin were not correlated.

**Table 7:** Agreement between the two tests on resistance isolate detection from the 63 isolates tested

Antimicrobial resistance isolates (%)	Resistance gene detected (%)	Agreement (%)
Erythromycin (n = 27; 42.8)	*ere(A)* (n = 0)	0
Gentamicin (n = 3; 4.8)	*aac(3)*-*IV* (n = 9; 14.3)	0
Streptomycin (n = 23; 36.5)	*aadA1* (n = 0)	0
Tetracycline (n = 30; 47.6)	*tet(A)* (n = 41; 65.1)	23 (76.7)
Sulfamethoxazole–trimethoprim (n = 22; 34.9)	*sul1* (n = 34; 54.0)	14 (63.6)
Chloramphenicol (n = 15; 23.8)	*catA1* (n = 5; 8.0)	2 (13.3)
	*cmlA* (n = 7; 11.1)	4 (26.7)

## Discussion

The magnitude of *E. coli* prevalence and its AMR patterns were investigated in raw meat samples of four livestock species at Addis Ababa and Bishoftu slaughter house. In our study, in raw meat, the prevalence of *E. coli* was 21.6%. The study indicated that *E. coli* was 2.8 times, 3.2 times, and 5.7 times more prevalent in chevon, mutton and chicken meat, respectively, than in beef meat. It implies that meat of livestock species types has a difference in magnitude of *E. coli* infection, therefore, has implications on the risk to public health. It has been reported that poor hygienic practices is responsible for contamination of meat by *E. coli* [20]. The magnitude of *E.* *coli* prevalence in this study was similar to the previous studies in different part of Ethiopia 22.2, 26.6 and 20.3% by Haileselassie et al. [21], Haimanot et al. [22] and Bitew et al. [23] respectively. However, Mekonen et al. [24] and Taye et al. [25] also reported higher prevalence of carcass contamination with an overall isolation rate of 40 and 30.97%, respectively. The current study deals with the magnitude of *E. coli* only at one point (i.e. slaughterhouse) of the food chain until it reaches the fork. It shouldn’t be overlooked that there could be ample chances for *E. coli* to infect the meat released from the slaughterhouse during transport, handling and processing chains before it reaches the consumer (fork) as hygienic practices are substandard in Ethiopia. It has been reported that poor hygienic practices is responsible for contamination of meat by *E. coli* [20].

In the current study, the prevalence of *E. coli* was significantly higher in chicken meat than in chevon, mutton and beef meat. This might be attributed to husbandry and production systems where, small holder farmers prefer to rear their chickens in backyard to scavenge. Thus, *E. coli* could be transmitted from intestinal normal flora of livestock to the chicken via the food chain [26]. Over all, the variations observed among the reported prevalence could be emanated from difference in hygiene, breed, geographical origins of animals and history of treatment with antimicrobial.

Emergence and dissemination of antimicrobial resistance is on the increase trend among enteric bacteria [27]. In this research the overall result showed that significantly high resistance rate to ampicillin (71.4%) and tetracycline (47.6%). More importantly, in the current report, chicken meat harbored the highest number of drug resistant *E. coli* isolates compared to the other meat origins. As expected, the most common resistance were found to older drugs such as ampicillin (Introduced in 1961) followed by tetracycline (Introduced in 1948) [28]. Similarly, Momtaz et al. [29] observed that tetracycline, sulfamethoxazole, chloramphenicol and trimethoprim resistance was the most common finding with the prevalence rate of 91.2, 45.6 and 29.8% respectively. Likewise, studies conducted in Korem, Ethiopia on diarrheic patients showed that the highest *E. coli* resistance were found against ampicillin followed by chloramphenicol, and tetracycline [30]. Hiko et al. [31] also found that the isolated *E. coli* is highly resistant to streptomycin, cephlaothin, tetracycline, ampicillin and trimethoprim. Another study revealed that all *E. coli* isolates from beef were found 100% resistance to ampicillin and amoxicillin and 33.33% to tetracycline [25]. The observed higher level of antimicrobial resistance might be attributed to the widespread and indiscriminate use of antibiotics in animals for medication and other prophylaxis purposes. Although tetracycline has the second most resistance in this study, it is one of the most commonly used antibiotics for the treatment of different infections, including *E. coli* in Ethiopia.

Recently, among gram negative bacteria, multidrug resistant (MDR) phenotypes are spreading widely [32].In the current study, the overall rate of multiple drug resistance was 46.0% and only 4.8% of the isolates were found sensitive to all antimicrobials tested (Table 4). This is in agreement with the findings of Schroeder et al. [33] and Zhao et al. [34] from USA that reported that multiple resistance to tetracycline, kanamycin, streptomycin, ampicillin, and sulfamethoxazole. Similarly, among sulfonamide resistant *E.* *coli* isolates, Wu et al. [35] reported that ampicillin and streptomycin are the two most frequently co-transferred resistance phenotypes. However, Daniel et al. [28] reported a resistance profile of tetracycline (80%) and streptomycin (74%), among the sulfonamide-resistant *E.* *coli* isolates. The transfer of resistance among microorganisms has long been recognized as a serious threat, contributing to the evolution and emergence of antibiotic resistant bacteria, thereby reducing the therapeutic potential against pathogens [36].

After phenotypic screening, genes associated with antimicrobial resistance was analyzed using PCR. In this study, the most commonly detected resistance genes were *blaCMY* (65.1%), *tetA* (65.1%) and *sul1* (54.0%)) for cephalosporin, tetracycline and sulfonamide respectively. However, streptomycin and erythromycin resistance genes were not detected. In Iran, among the *E. coli* isolates, 52.6% of resistance to tetracycline as well as 47.4% for both sulfonamides and erythromycin was reported [31]. The prevalence of different resistance genes from pediatric patient *E. coli* isolate were also reported 85.06, 60.38, 57.79, 90.25, 40.25 and 54.54% positive for *tetA*, *cmlA*, *SHV β*-*lactamase*, *CITM*, *sul1* and *aac*(*3*)-*IV* resistance genes respectively [37].

The phenotypic and genotypic resistance patterns with in the same *E. coli* isolates might be strongly correlated [38]. In our research, detection of tetracycline resistance by disk diffusion test and identification of its resistance gene *tet*(*A*) by PCR test, sulfamethoxozole–trimethoprim and *sulI,* chloramphenicol and *cmlA* were strongly associated. But there was a lack of association between phenotypic erythromycin resistance and molecular detection of *ere*(*A*), streptomycin and *aadA1,* chloramphenicol and *catA1* resistance gene. Similarly, reports from Thailand showed that, *E. coli* isolates from chickens were found to be resistant to tetracycline (90%) and erythromycin (73.3%) in Agar disk diffusion assays and these resistance properties were associated with *tet*(A) and *ere*(A) resistance genes respectively [4]. However, the presence of resistance phenotypes might not represent all the underlying resistance genes. Alternatively, the presence or absence of a resistance gene might not indicate the particular isolate is resistant or susceptible to an antimicrobial [39].

The observed discrepancy in the resistance level of genotypes and phenotypes might be attributed to either of or combination of the reasons including but not limited to the presence of unexpressed genes in the bacterial isolates, not containing the possible resistance genes in the test, use of incorrect cut points of test results used for resistance and susceptibility classification, or some of resistance phenotypes are caused by due to point mutations rather than gene transfer or acquisition [40].

## Conclusions

This study revealed that raw meat available for consumers in Ethiopia was often contaminated with *E. coli*. We reported on a comprehensive study of the phenotypic and molecular basis for Multi Drug Resistance (MDR) in meat *E. coli* isolates recovered from Ethiopian Abattoir. Furthermore, a high rate of resistance to Ampicillin and resistance to more than one class of antibiotics among *E. coli* isolates was found. Additionally, several strains were found positive for the *bla*_CMY_, *tet*(*A*), and *sul1* antimicrobial resistance genes. All these findings suggest that the consumption of undercooked meat or food cross-contaminated with *E. coli* may pose a serious threat to consumer health.

## Abbreviations

AMR: antimicrobial resistance
MDR: multidrug resistance
*aadA1*: streptomycin resistance gene
*tet(A)*: tetracycline resistance gene
*aac(3)*-*IV*: gentamycin resistance gene
s*ulI*: sulphanamide resistance gene
*blaSHV*: betalactam resistance gene (pencillin)
*blaCMY*: betalactam resistance gene (cephalosporin)
*ere(A)*: erythromycin resistance gene
*catA1*: chloramphenicol resistance gene
c*mlA*: chloramphenicol resistance gene
PCR: polymerase chain reaction
MgCl_2_: magnesium chloride
dNTP: deoxynucleotide tri phosphate
UV: ultra violet
Bp: base pair
OR: odds ratio
DNA: deoxynucleic acid
SPSS: statistical package for social science
χ2: Pearson chi square
P value: predictive value
R: resistance
I: intermediate
S: susceptible
E: erythromycin
Amp: ampicillin
GN: gentamycin
S: streptomycin
TE: tetracycline
Sxt: sulfamethoxazole–trimethoprim
C: chloramphenicol
ISO: International Organization for Standardization
CLIS: Clinical and Laboratory Standards Institute
